# Supplementary material for: Impact of superior mesenteric artery (SMA) adherent tissue in the context of pancreatic head resections- an observational study
Source: BMC Cancer. 2026 Jun 24;26:771. doi: 10.1186/s12885-026-16278-7 (PMC13292570; doi:10.1186/s12885-026-16278-7)
Supplement: Supplementary file 1 — Supplementary Material 1. [file 12885_2026_16278_MOESM1_ESM.docx]

**STROBE Checklist**

*Manuscript: Impact of superior mesenteric artery (SMA) adherent tissue in the context of pancreatic head resections- an observational study*

Journal: BMC Cancer | Date: April 2026

| **Section/Topic** | **No** | **Recommendation & How Addressed in This Manuscript** | **Page/Para** |
| --- | --- | --- | --- |
| **TITLE AND ABSTRACT** | | | |
| Title and abstract | 1 | (a) Indicate the study's design with a commonly used term in the title or the abstract.  "Impact of superior mesenteric artery (SMA) adherent tissue in the context of pancreatic head resections" — Study design (prospective cohort) now added to title per Response to Editor Comment 1.1.  b) Provide an informative and balanced summary of what was done and what was found.  Abstract revised to explicitly state semi-circumferential SMA-adjacent specimen analysis; findings now described as hypothesis-generating (Response Comment 3.5). | Title, Abstract |
| **INTRODUCTION** | | | |
| Background/rationale | 2 | Explain the scientific background and rationale for the investigation being reported.  Introduction reorganized into 3 paragraphs: (1) historical development of pancreatic head resection / TRIANGLE operation; (2) clinical problem of postoperative diarrhea and its impact on adjuvant therapy; (3) rationale and aims of the present study (Response Comment 3.6). | Introduction |
| Objectives | 3 | State specific objectives, including any prespecified hypotheses. Study objective is the systematic prospective histopathological quantification of malignant cell involvement in SMA-adjacent tissue in resectable PDAC. Stated in revised Introduction. | Introduction |
| **METHODS** | | | |
| Study design | 4 | Present key elements of study design early in the paper.  Prospective cohort study. Design now included in title (Editor Comment 1.1). Semi-circumferential SMA-adjacent tissue harvested at time of pancreatic head resection. | Methods |
| Setting | 5 | Describe the setting, locations, and relevant dates, including periods of recruitment, exposure, follow-up, and data collection. Department of General and Visceral Surgery, University Medical Centre Göttingen. Prospective recruitment dates and follow-up periods specified in Methods. | Methods |
| Participants | 6 | Cohort study: Give the eligibility criteria, and the sources and methods of selection of participants. Describe methods of follow-up.  n=66 patients with primarily resectable pancreatic cancer undergoing pancreatic head resection. Eligibility criteria, exclusion criteria, and follow-up method described in Methods. | Methods |
| Variables | 7 | Clearly define all outcomes, exposures, predictors, potential confounders, and effect modifiers. Give diagnostic criteria if applicable.  Primary outcome: SMA involvement (SMA+ vs. SMA−). Confounding by R-status explicitly acknowledged (Response Comment 3.7). Tumor histology, margin status (R0/R1), perineural invasion (Pn1) defined. | Methods |
| Data sources / measurement | 8 | For each variable of interest, give sources of data and details of methods of assessment.  SMA-adjacent tissue entirely embedded, serially sectioned at 3–5 μm. H&E staining; IHC (CK7, CK19, CK20) in equivocal cases. R-status per current guidelines (R1: tumour cells ≤1 mm margin). One dedicated pathologist, Dept. of Pathology, UMG (Response Comment 3.3). | Methods |
| Bias | 9 | Describe any efforts to address potential sources of bias.  Formal blinding of pathologists to clinical outcomes not performed — acknowledged as limitation. Semi-circumferential (not full circumferential) approach may underestimate true SMA+ rate — stated as limitation (Response Comment 5.1). | Methods / Limitations |
| Study size | 10 | Explain how the study size was arrived at.  No a priori sample size calculation was performed given prospective exploratory design and low expected incidence of SMA involvement. Explicitly stated as limitation in Statistics section (Response Comment 5.2). | Methods / Statistics |
| Quantitative variables | 11 | Explain how quantitative variables were handled in the analyses. If applicable, describe which groupings were chosen and why.  Age reported as median (range). Continuous variables dichotomised (SMA+/−; R0/R1) for survival analysis. Formatting standardized: '70 (48–87)' (Response Comment 5.11). | Methods / Table 1 |
| Statistical methods | 12 | Cohort study: (a) Describe all statistical methods, including those used to control for confounding. (b) Describe any methods used to examine subgroups and interactions.  Dedicated Statistical Analysis subsection added. Kaplan-Meier survival analysis; log-rank test; HR with 95% CI reported; p<0.05 significance threshold; GraphPad Prism v10.0. No multivariable Cox regression — insufficient events (n=5 SMA+); stated as limitation. (Response Comments 3.4, 5.3, 5.12). | Methods / Statistics |
| **RESULTS** | | | |
| Participants | 13 | Report numbers of individuals at each stage of study (e.g. numbers potentially eligible, examined, confirmed eligible, included in the study, completing follow-up, and analysed). Give reasons for non-participation at each stage.  n=66 patients included. SMA+ n=5 (7.6%), SMA− n=61. Numbers consistent throughout manuscript (Response Comment 3.7). Patients with benign lesions or preoperatively categorized with AMS-Infiltration in CT/MRI were excluded. | Results |
| Descriptive data | 14 | Cohort study: Give characteristics of study participants (e.g. demographic, clinical, social) and information on exposures and potential confounders.  Table 1 presents baseline characteristics including age, sex, tumour histology, T-stage, N-stage, R-status, Pn status. Formatting corrected throughout (Response Comment 5.11). | Results / Table 1 |
| Outcome data | 15 | Cohort study: Report numbers of outcome events or summary measures over time.  SMA+ rate 7.6% (n=5/66), exclusively in PDAC. R1 in 4/5 SMA+ cases. Median OS reported per group; HR with 95% CI and exact p-values from log-rank test included in Results text (Response Comment 5.9). | Results |
| Main results | 16 | Give unadjusted estimates and, if applicable, confounder-adjusted estimates. Report category boundaries when continuous variables were categorized.  Unadjusted Kaplan-Meier curves presented for SMA+/− and R0/R1 subgroups. No adjusted estimates (multivariable Cox not feasible with n=5; stated as limitation). Survival differences between SMA+ and SMA− strongly confounded by R-status; explicitly stated (Response Comment 3.7). | Results / Discussion |
| Other analyses | 17 | Report other analyses done — e.g. analyses of subgroups and interactions, and sensitivity analyses.  Subgroup analysis SMA+/R1 vs. SMA−/R1 vs. SMA−/R0. Direct SMA+/R0 vs. SMA−/R0 comparison not performed (only n=1 in SMA+/R0 group). Stated that all survival data from small subgroups are hypothesis-generating (Response Comment 5.2). | Results |
| **DISCUSSION** | | | |
| Key results | 18 | Summarise key results with reference to study objectives.  7.6% SMA involvement rate (exclusively PDAC). Semi-circumferential dissection. R-status as dominant survival determinant. Findings are hypothesis-generating (Response Comment 3.5). | Discussion |
| Limitations | 19 | Discuss limitations of the study, taking into account sources of potential bias or imprecision. Discuss both direction and magnitude of any potential bias.  (1) Small n (n=5 SMA+) → underpowered survival analyses; (2) No formal blinding of pathologists; (3) Semi-circumferential (180°) — may underestimate true incidence; (4) No a priori sample size calculation; (5) No multivariable Cox regression; (6) No diarrhea/adjuvant therapy data collected in this cohort; (7) R-status confounding (Response Comments 3.3, 3.4, 5.1–5.6). | Discussion |
| Interpretation | 20 | Give a cautious overall interpretation of results considering objectives, limitations, multiplicity of analyses, results from similar studies, and other relevant evidence.  Findings contextualized with TRIANGLE procedure literature; comparison with CME in colorectal surgery (Luzon et al., Surg Endosc 2022). Adjuvant therapy/diarrhea discussed with external references only (Thorsen et al.). R-status as primary survival driver; SMA+ as potential aggressiveness marker (Response Comments 3.8, 4.1, 5.4–5.8). | Discussion |
| Generalisability | 21 | Discuss the generalisability (external validity) of the study results. Results limited to patients with primarily resectable malign tumors of the pancreas/distal bile duct undergoing semi-circumferential SMA dissection at a single centre. Non-PDAC histologies (small n) preclude generalisation. Future studies should stratify by PDAC exclusively; or present a lager cohort to verify that e.g. distal bile duct carcinoma do not infiltrate the tissue surrounding AMS. These patients may benefit from a rther less radical resection. Multicenter validation recommended (Response Comment 3.8). | Discussion |
| **OTHER INFORMATION** | | | |
| Funding | 22 | Give the source of funding and the role of the funders for the present study and, if applicable, for the original study on which the present article is based.  No funding | Declarations |
