# Supplementary material for: Impact of superior mesenteric artery (SMA) adherent tissue in the context of pancreatic head resections- an observational study
Source: BMC Cancer. 2026 Jun 24;26:771. doi: 10.1186/s12885-026-16278-7 (PMC13292570; doi:10.1186/s12885-026-16278-7)
Supplement: Supplementary file 2 — Supplementary Material 2. [file 12885_2026_16278_MOESM2_ESM.docx]

**Supplementary Table S1**

| **Basic clinical data (n=66 patients)** |  |  |
| --- | --- | --- |
|  | **SMA-positive (n=5)** | **SMA-negative (n=61)** |
| **Age (years)** | 63 years (Median) | 73 years (Median) |
| **Sex**  Male  Female | n=4  n=1 | n=29  n=32 |
| **Tumour entity**  PDAC | n=5 | n=34 |
| Distal bile duct cancer | n=0 | n=14 |
| Duodenal adenocarcinoma | n=0 | n=1 |
| Acinar cell carcinoma | n=0 | n=1 |
| Periampullary carcinoma | n=0 | n=9 |
| Neuroendocrine tumour | n=0 | n=1 |
| Colloid carcinoma | n=0 | n=1 |
| **T stage**  pT1  pT2  pT3 | n=1  n=0  n=1 | n=8  n=27  n=25 |
| **Nodal status**  positive  negative | n=4  n=1 | n=46  n=15 |
| **Distant metastasis**  cM0  cM1 | n=5  n=0 | n=60  n=1 |
| **Lymphovascular invasion**  L0  L1  LX | n=3  n=1  n=1 | n=23  n=35  n=3 |
| **Venous invasion**  V0  V1  VX | V0  n=4  n=0  n=1 | 45 (68.2%)  n=41  n=16  n=3 |
| **Perineural invasion**  Pn0  Pn1  PnX | n=0  n=4  n=1 | n=13  n=45  n=3 |
| **Circumferential resection margin**  CRM positive  CRM negative  CRM not assessable | n=2  n=2  n=1 | n=25  n=22  n=14 |
| **Grading**  G1  G2  G3  GX | n=0  n=2  n=2  n=1 | n=3  n=41  n=16  n=1 |
| **Residual tumour**  R0  R1 | n=2  n=3 | n=53  n=8 |
| **Neoadjuvant treatment**  yes  no | n=1  n=4 | n=4  n=57 |
