# Supplementary material for: Impact of superior mesenteric artery (SMA) adherent tissue in the context of pancreatic head resections- an observational study
Source: BMC Cancer. 2026 Jun 24;26:771. doi: 10.1186/s12885-026-16278-7 (PMC13292570; doi:10.1186/s12885-026-16278-7)
Supplement: Supplementary file 3 — Supplementary Material 3. [file 12885_2026_16278_MOESM3_ESM.docx]

**Impact of superior mesenteric artery (SMA) adherent tissue in the context of pancreatic head resections- an observational study.**

Azadeh Azizian^1,2^, Markus Bernhardt^1,2^, Felix Rühlmann^1,2^, Stina Schild-Suhren^1^, Marie Crede^1^, Florian Bösch^1^, Daniel Gärtner^2^, Michael Ghadimi^1^, Jochen Gaedcke^1,2^*

^1^ Department of General, Visceral, and Paediatric Surgery, University Medical Centre Göttingen, Göttingen, Germany

^2^ Department of General and Visceral Surgery, Städtisches Klinikum Karlsruhe, Karlsruhe, Germany

*Correspondence: jochen.gaedcke@klinikum-karlsruhe.de

# Abstract

**Background:** Resection of the periadventitial neurolymphatic tissue around the superior mesenteric artery (SMA) is considered a crucial component of oncologically complete resection in pancreatic head and corpus carcinomas. However, a substantial proportion of patients experience severe postoperative diarrhea due to resection of the splanchnic nerves within the SMA-adjacent tissue. The aim of the present study was to histopathologically analyze the semi-circumferential periadventitial neurolymphatic tissue surrounding the SMA in pancreatic cancer patients for the presence of malignant cells, in order to evaluate its oncological significance and to determine whether its resection may confer a long-term survival benefit.

**Methods:** A total of 66 patients with resectable malignant tumours of the pancreas treated at the University Medical Centre Göttingen were prospectively enrolled in this exploratory pilot study. The dissected semi-circumferential tissue around the SMA was processed by complete embedding and serial sectioning and analyzed for the presence of malignant cells. Survival analyses were performed using the Kaplan–Meier method with log-rank testing.

**Results:** In 7.6% of all cases (n=5), malignant cells were found in the tissue adjacent to the SMA (SMA^+^). In all SMA^+^-patients, histopathological workup showed a pancreatic ductal adenocarcinoma (PDAC). In 80% of SMA^+^-patients (n=4), an R1 resection margin was confirmed. Patient outcomes (overall survival [OS] and cancer-specific survival [CSS]) were primarily determined by R-status.

**Conclusions:** Malignant SMA-adjacent tissue involvement was identified in 7.6% of resectable pancreatic head tumours and was exclusive to PDAC. R-status appears to be the dominant determinant of survival. These findings should be considered hypothesis-generating and require validation in larger prospective cohorts.

**Keywords:** Pancreatic cancer; superior mesenteric artery; TRIANGLE technique; pancreaticoduodenectomy; periadventitial dissection

# Background

Surgical therapy for pancreatic cancer has evolved continuously over the last decades. From the first surgical resection by Alfred Kausch in 1909, through the pylorus-preserving modification by Longmire and Traverso in 1977, to the Heidelberg technique introduced in 2019 – also known as the TRIANGLE Operation [1]. Schneider et al. presented data from patients initially considered borderline resectable who underwent pseudoneoadjuvant chemotherapy; by performing periadventitial dissection in the area of the SMA, this technique allowed complete tumor removal in a relevant number of patients without arterial resection and with improved long-term outcomes. This approach was further developed into a complete tissue clearance between the SMA, portal vein, and common hepatic artery without increasing perioperative mortality [2].

As reported by Hang et al., resection of the entire periadventitial neurolymphatic tissue around the SMA may result in a higher rate of postoperative diarrhea [3]. Lei et al. similarly examined different extents of neurolymphatic tissue resection in a single-centre series, comparing operative outcomes and survival across approaches of varying radicality [4]. This may result in electrolyte imbalances, significant fluid losses, impaired quality of life, and reduced capacity to complete adjuvant chemotherapy.

To further investigate whether resection of the periadventitial tissue around the SMA is necessary in patients with primarily resectable pancreatic cancer, we examined a representative tissue specimen from one side of the SMA for the presence of malignant cells. The present study was designed as a prospective, exploratory, single-centre pilot analysis. Accordingly, no a priori sample size calculation was performed, and the findings should be interpreted as hypothesis-generating. We aimed to assess the frequency of malignant SMA involvement and its potential association with long-term survival outcomes.

# Methods

## Study design and patient selection

This was a prospective, exploratory, single-centre pilot study conducted at the Department of General, Visceral, and Paediatric Surgery, University Medical Centre Göttingen, Germany. All patients planned for pancreatic head resection between 2022 and 2024 were screened for eligibility. Inclusion criteria required a resectable tumour of the pancreatic head with tumour mass clearly distant from the portal vein and the SMA on preoperative imaging. Patients with benign lesions confirmed on final histopathological examination were excluded from further analysis.

## Surgical technique

Pancreatic head resection was performed as previously described. In patients with a clearly localized tumour in the pancreatic head situated at a safe distance from the mesenteric artery, the tissue surrounding the SMA was resected semi-circumferentially along the mesopancreas. Semi-circumferential dissection was defined as resection of approximately 180° of the periadventitial tissue, encompassing the right and ventral aspects of the SMA (corresponding to the 9 o’clock to 3 o’clock orientation), which represents the anatomical zone most closely related to the pancreatic head and the mesopancreas. The portal vein was held laterally to the pancreatic body (Figure 1A), and medial rotation of the pancreatic head exposed the SMA. Following resection of the pancreatic head, the remaining tissue adherent to the SMA was dissected (Figure 1B and 1C). Histopathological analysis of the dissected tissue was performed separately (Figure 1D).


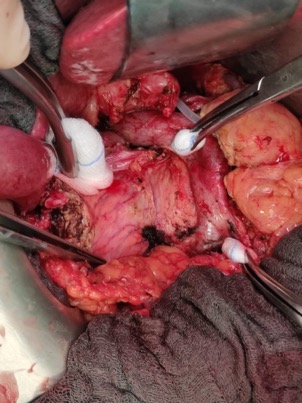

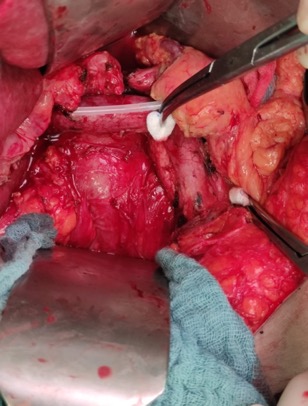


**B**

**A**


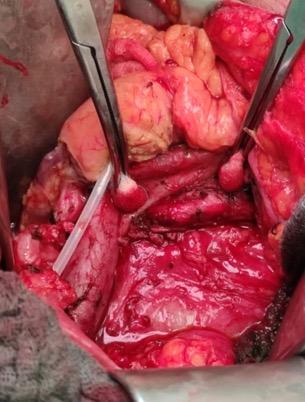

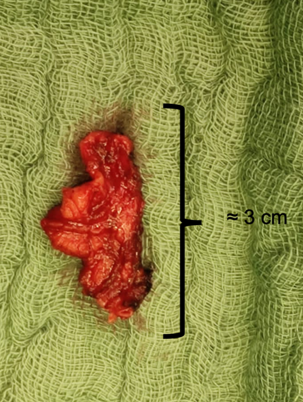


**D**

**C**

**Figure 1A:** Portal vein and SMA prior to the resection of the pancreatic head. Blue arrow marks the portal vein, orange arrow marks the SMA. **Figure 1B and 1C:** SMA after the semi-circumferential resection of the surrounding tissue. **Figure 1D:** Tissue dissected from the SMA.

## Histopathological workup

The resected SMA-adjacent tissue specimens were processed according to standard pathological protocols. Each specimen was entirely embedded and serially sectioned at 3–5 μm intervals. Sections were stained with haematoxylin and eosin (H&E). In cases where morphological assessment was inconclusive, immunohistochemical staining was performed using standard epithelial markers, including cytokeratin 7 (CK7), cytokeratin 19 (CK19), and cytokeratin 20 (CK20), to confirm or exclude the presence of malignant cells. Each specimen was evaluated by one dedicated pathologist at the Department of Pathology, University Medical Centre Göttingen. The resection margin (R-status) was defined according to current guidelines: R0 was defined as no tumour cells at the resection margin, and R1 as tumour cells within 1 mm of the resection margin.

## Statistical analysis

All statistical analyses were performed using GraphPad Prism (version 10.0; GraphPad Software, San Diego, CA, USA). Survival curves were generated using the Kaplan–Meier method, and differences between groups were assessed using the log-rank test. Results are reported with exact p-values; a two-sided p-value of <0.05 was considered statistically significant. Hazard ratios (HR) with 95% confidence intervals (CI) were calculated where applicable. Due to the prospective, exploratory nature of this study and the low overall incidence of SMA-positive cases, no a priori sample size calculation was performed. This represents a recognised limitation of the present analysis, and the survival data derived from the small SMA⁺ subgroup (n=5) should therefore be interpreted as hypothesis-generating rather than confirmatory.

# Results

## Patient characteristics

A total of 70 patients met the inclusion criteria, with tumour mass clearly distant from the portal vein and SMA. In four cases, the pancreatic lesion proved to be benign on histopathological examination and was excluded from further analysis, leaving 66 patients with malignant lesions for analysis. Clinical and pathological data are summarised in Table 1.

**Table 1: Clinical characteristics of all enrolled patients with malignant pancreatic lesions (n=66)**

| **Basic clinical data (n=66 patients)** |  |  |
| --- | --- | --- |
| Age (years) | Median (min–max) | 70 (48–87) |
| Sex | Male | 33 (50%) |
|  | Female | 33 (50%) |
| Type of surgery | Traverso–Longmire | 62 (94.0%) |
|  | Kausch–Whipple | 1 (1.5%) |
|  | Total pancreatectomy | 3 (4.5%) |
| Tumour entity | PDAC | 39 (59.0%) |
|  | Distal bile duct cancer | 14 (21.2%) |
|  | Duodenal adenocarcinoma | 1 (1.5%) |
|  | Acinar cell carcinoma | 1 (1.5%) |
|  | Periampullary carcinoma | 9 (14.3%) |
|  | Neuroendocrine tumour | 1 (1.5%) |
|  | Colloid carcinoma | 1 (1.5%) |
| T stage | pT1 | 9 (13.6%) |
|  | pT2 | 27 (41.0%) |
|  | pT3 | 26 (39.3%) |
| Nodal status | Positive | 50 (76.0%) |
|  | Negative | 16 (24.2%) |
| Distant metastasis | cM0 | 65 (98.5%) |
|  | cM1 | 1 (1.5%) |
| Lymphovascular invasion | L0 | 26 (39.3%) |
|  | L1 | 36 (55.0%) |
|  | LX | 4 (6.0%) |
| Venous invasion | V0 | 45 (68.2%) |
|  | V1 | 16 (24.2%) |
|  | VX | 4 (6.0%) |
| Perineural invasion | Pn0 | 13 (20.0%) |
|  | Pn1 | 49 (74.0%) |
|  | PnX | 4 (6.0%) |
| Circumferential resection margin | CRM positive | 27 (41.0%) |
|  | CRM negative | 24 (36.3%) |
|  | CRM not assessable | 15 (23.0%) |
| Grading | G1 | 3 (4.5%) |
|  | G2 | 43 (65.2%) |
|  | G3 | 18 (27.2%) |
|  | GX | 2 (3.0%) |
| Residual tumour | R0 | 55 (83.3%) |
|  | R1 | 11 (16.7%) |
| Neoadjuvant treatment | Yes | 5 (7.6%) |
|  | No | 61 (92.4%) |

*PDAC pancreatic ductal adenocarcinoma; CRM circumferential resection margin; R0 no tumour cells at resection margin; R1 tumour cells within 1 mm of resection margin*

## Analysis of the adherent tissue around the SMA

## Survival data

## Figure 2A illustrates overall survival (OS) of all patients dependent on the state of the adherent tissue of SMA (malignant cells detected in the adherent tissue around SMA, SMA^+^; no malignant cells detected in the adherent tissue around SMA, SMA^-^). Median overall survival for SMA^+^ patients was 11.01 months versus 26.14 months in SMA^-^ patients (HR: 11.24; 95% CI: 1.803-70.08; p-value 0.0116 Gehan-Breslow-Wilcoxon test and p-value: 0.0096 logrank test)

Figure 2B shows OS of all patients with PDAC dependent on their SMA state. Median overall survival was 11.01 months in SMA^+^ patients versus 25.84 months in SMA^-^ PDAC patients (HR: 14.54; 95% CI: 2.161-97.88; p-value 0.0051 Gehan-Breslow-Wilcoxon test and p-value: 0.0059 logrank test). Figure 2C shows OS depending on their resection margin status (R0 vs. R1). Median overall survival was 12.39 months in R1-resected patients versus 29.19 months in R0-resected patients (HR: 21.56; 95% CI: 5.144-90.39; p-value 0.0002 Gehan-Breslow-Wilcoxon test and p-value: <0.0001 logrank test). Finally, Figure 2D shows all mentioned groups combined.

##
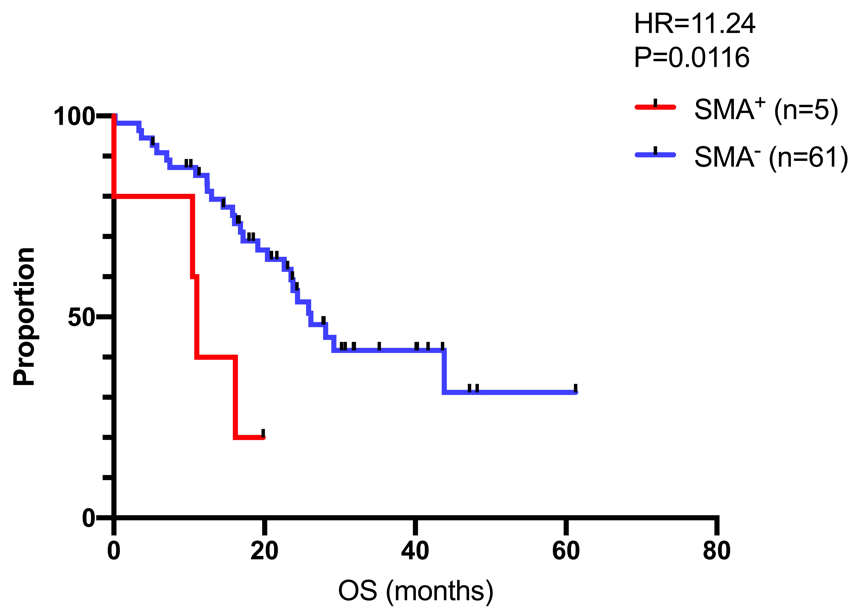


**Figure 2A**


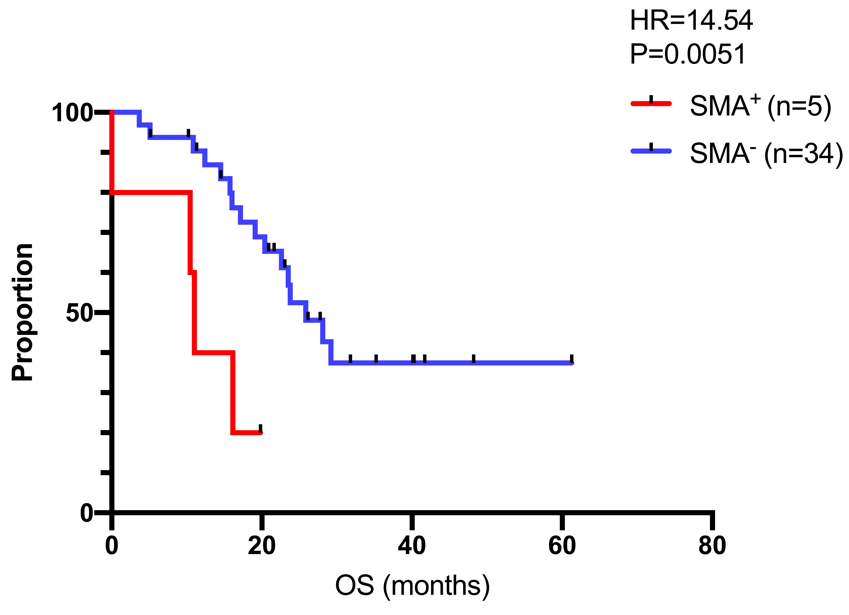


**Figure 2B**


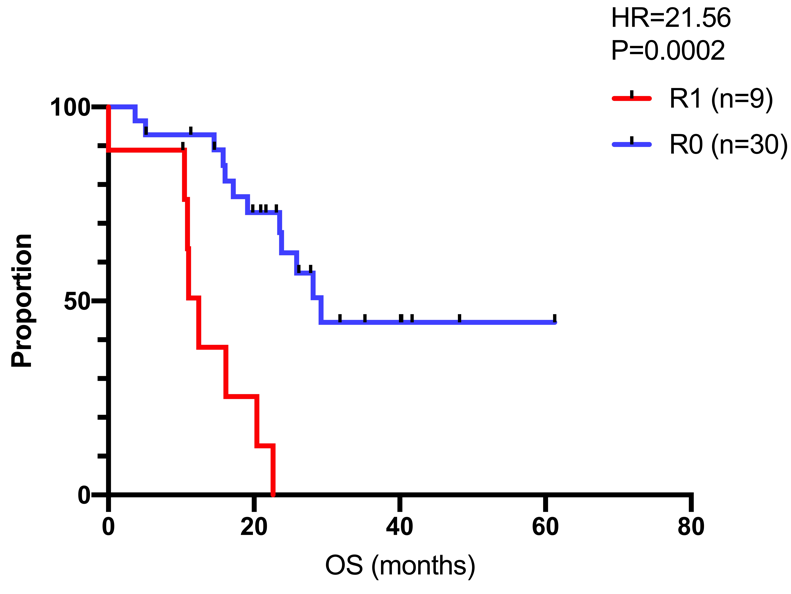


**Figure 2C**


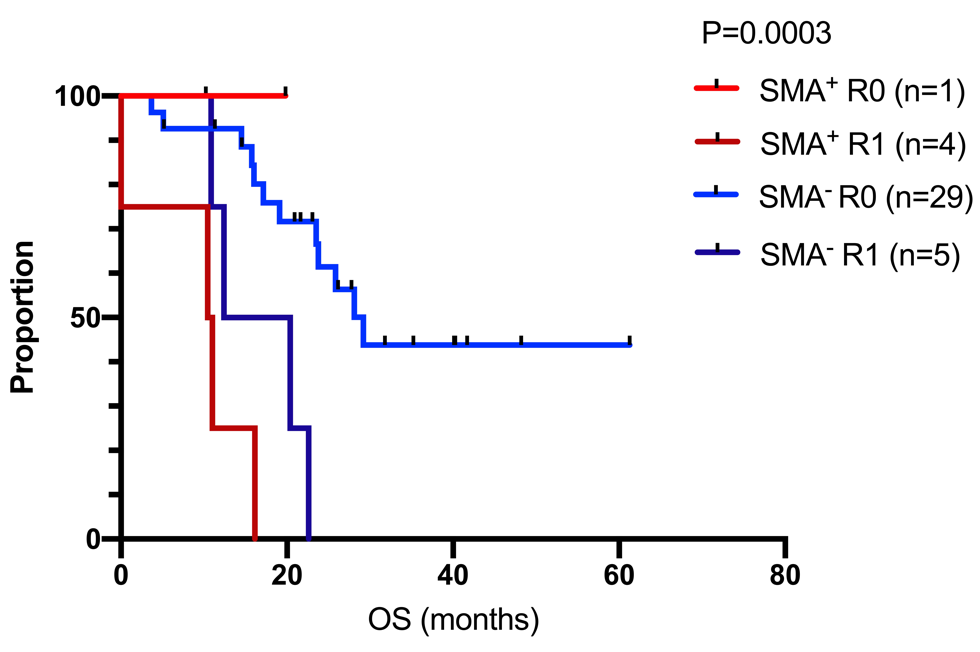


**Figure 2D**

## Figure 2: Kaplan-Meier-analysis for overall survival of the cohort. 2A: Comparison between all patients with malignant cells in the adherent tissue around SMA, SMA^+^, and all patients with no malignant cells in the adherent tissue around SMA, SMA^-^. The difference in OS is significantly higher in SMA^-^ (p=0.0116). Median overall survival for SMA^+^ patients was 11.01 months versus 26.14 months in SMA^-^ patients (HR: 11.24; 95% CI: 1.803-70.08; p-value 0.0116 Gehan-Breslow-Wilcoxon test and p-value: 0.0096 logrank test)

**2B:** Comparison between all PDAC patients with malignant cells in the adherent tissue around SMA, SMA^+^, and all patients with no malignant cells in the adherent tissue around SMA, SMA^-^. The difference in OS is significantly higher in SMA^-^ (p=0.0051). Median overall survival was 11.01 months in SMA^+^ patients versus 25.84 months in SMA^-^ PDAC patients (HR: 14.54; 95% CI: 2.161-97.88; p-value 0.0051 Gehan-Breslow-Wilcoxon test and p-value: 0.0059 logrank test) **2C:** Comparison between all PDAC patients with free resection margins, R0, and PDAC patients with tumour cells at resection margin, R1 (p=0.0002). Median overall survival was 12.39 months in R1-resected patients versus 29.19 months in R0-resected patients (HR: 21.56; 95% CI: 5.144-90.39; p-value 0.0002 Gehan-Breslow-Wilcoxon test and p-value: <0.0001 logrank test) **2D** shows all combined groups (SMA^+^R0, SMA^+^R1,SMA^-^R0, and SMA^-^R1), significant difference was seen in SMA^-^R0 vs SMA^-^R1, p=0.0003).

Figure 3A accordingly shows disease-free survival (DFS) of all patients diagnosed with PDAC, Figure 3B shows cancer-specific survival of the same cohort.


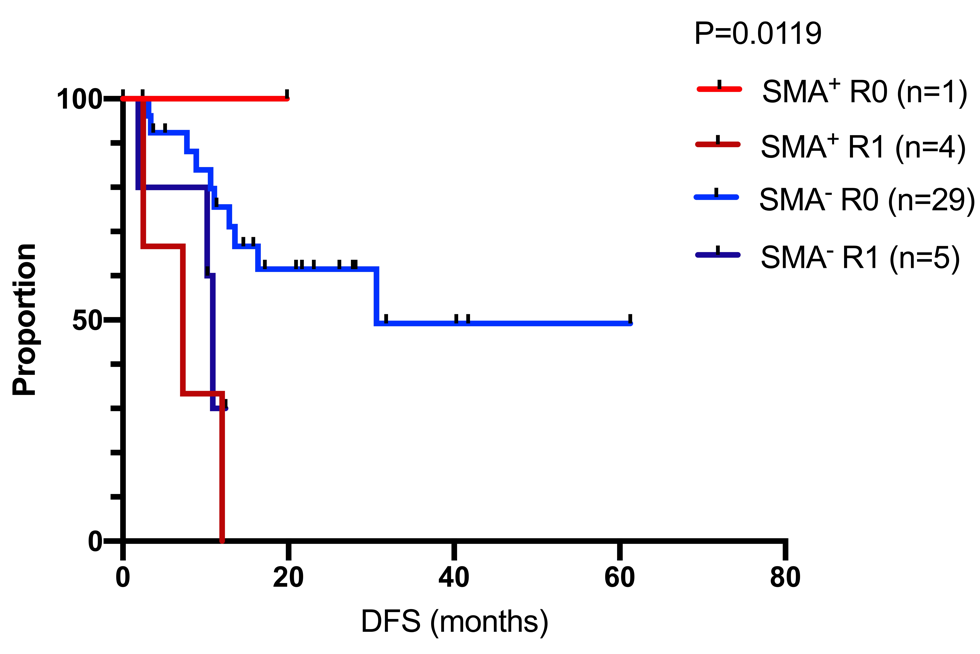


**Figure 3A**


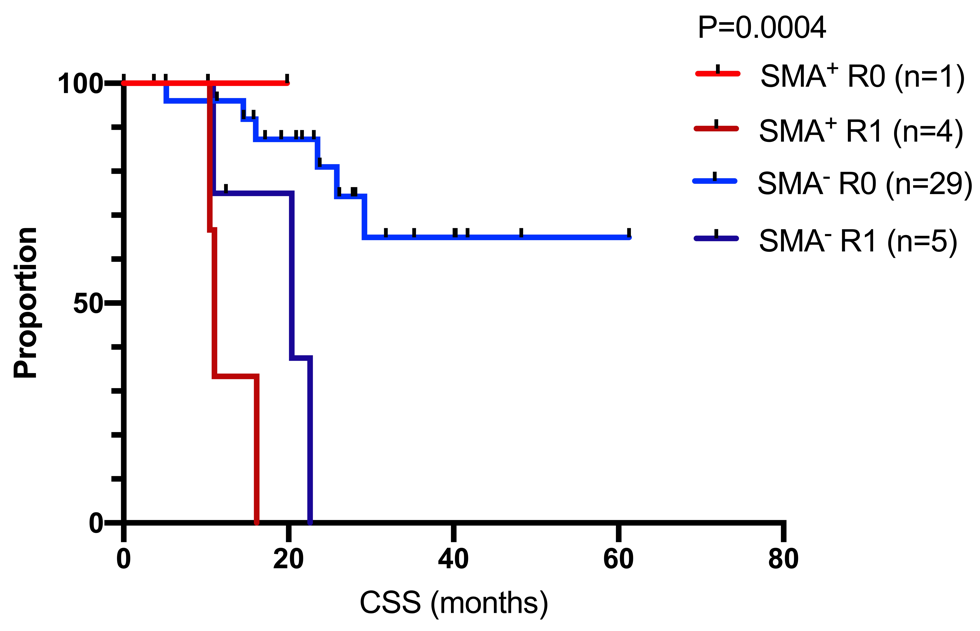


**Figure 3B**

***Figure 3****:* Disease-free survival and Cancer-specific survival of all enrolled patients with histopathologically confirmed PDAC. **A:** Significant difference in DFS was seen in SMA^-^R0 vs SMA^-^R1, p=0.0119. B: Significant difference in CSS was seen in SMA^-^R0 vs SMA^-^R1, p=0.0004.

# Discussion

These findings provide a plausible biological explanation for why the TRIANGLE procedure, when applied indiscriminately to all patients with resectable PDAC, does not consistently translate into a measurable survival benefit: in the majority of patients, the SMA-adjacent tissue is histopathologically free of tumour cells, and therefore routine radical dissection does not alter the oncological outcome but exposes patients to the risk of severe postoperative diarrhea.

Hang et al. similarly confirmed the safety of the TRIANGLE procedure, while reporting no survival benefit for patients with resectable PDAC [3].

Lei et al. compared three approaches to neurolymphatic tissue resection of varying radicality in a single-centre study, demonstrating comparable operative times, complication rates, and postoperative hospital stays [4]. The more radical resection groups showed improved time-to-progression rates, yet overall survival did not differ significantly.

An analogous discussion has taken place in colorectal surgery, where complete mesocolic excision (CME) has been associated with improved oncological outcomes in colon cancer [5]. The concept of defined plane dissection with systematic lymphadenectomy parallels the rationale for periadventitial SMA dissection in pancreatic surgery, and comparative data from CME cohorts may inform future study design.

Severe postoperative diarrhea may preclude or delay adjuvant chemotherapy. The incidence of this complication remains debated but is plausibly related to the extent of periadventitial dissection. In some cases, symptomatic management may be required on a long-term basis. Data from comparable CME colectomy cohorts have demonstrated that postoperative functional outcomes can influence adjuvant therapy completion rates [6]. In the present study, data on postoperative diarrhea and adjuvant chemotherapy completion were not systematically collected; this should be addressed in future studies.

Notably, Chen et al. demonstrated that the TRIANGLE operation combined with adequate adjuvant chemotherapy was associated with significantly lower recurrence rates, potentially improving survival in patients able to tolerate postoperative systemic therapy [7]. Therefore, the necessity of routine resection of the SMA-adjacent tissue in all patients warrants careful consideration, as postoperative quality of life and functional recovery are critical determinants of eligibility for intensive adjuvant chemotherapy.

Based on the present findings, we propose the following framework for clinical decision-making: in patients with resectable PDAC, the SMA region should be carefully assessed intraoperatively. If involvement is suspected, aggressive periadventitial dissection to achieve R0 margins is warranted; this must be carefully balanced against the risk of postoperative diarrhea and its potential impact on adjuvant chemotherapy completion. For patients with non-PDAC histology and clearly resectable tumours, the present evidence does not support routine SMA tissue resection, pending larger confirmatory series.

**Limitations**

The present study has several limitations that should be acknowledged. First, the pilot study was designed as a prospective, exploratory, single-centre analysis; accordingly, no a priori sample size calculation was performed, and the findings must be interpreted as hypothesis-generating rather than confirmatory. Second, the number of SMA-positive cases (n=5, 7.6%) is small, which limits the statistical power of subgroup comparisons and survival analyses within this group. Third, data on postoperative diarrhea were not systematically collected, which precludes a formal assessment of the functional morbidity associated with periadventitial dissection. Similarly, information on adjuvant chemotherapy completion rates was not available; this is of particular relevance given that postoperative diarrhea may compromise the ability to complete systemic therapy and thereby influence long-term outcomes. These limitations should be addressed in future prospective studies with adequate sample sizes and systematic collection of functional and oncological outcome data.

# List of Abbreviations

CRM: circumferential resection margin; CSS: cancer-specific survival; DFS: disease-free survival; H&E: haematoxylin and eosin; HR: hazard ratio; IHC: immunohistochemistry; OS: overall survival; PDAC: pancreatic ductal adenocarcinoma; SMA: superior mesenteric artery

# Declarations

## Ethics approval and consent to participate

The study was conducted in accordance with the Declaration of Helsinki. Ethical approval was obtained from the Institutional Review Board of the University Medical Centre Göttingen. Written informed consent was obtained from all patients included in the study.

## Consent for publication

Not applicable.

## Availability of data and materials

The datasets used and/or analysed during the current study are available from the corresponding author upon reasonable request.

## Competing interests

The authors declare that they have no competing interests.

## Funding

This research received no external funding.

## Authors' contributions

Conceptualization: A.A. and J.G.; methodology: A.A., M.B., F.R.; formal analysis: A.A., M.B.; investigation: A.A., M.B., F.R., S.S., M.C., F.B., D.G.; resources: M.G. and J.G.; data curation: A.A. and J.G.; writing – original draft preparation: A.A.; writing – review and editing: M.B., F.R., M.G., J.G.; supervision: M.G. and J.G.; project administration: J.G. All authors read and approved the final manuscript.

## Acknowledgements

The authors thank the staff of the Department of Pathology, University Medical Centre Göttingen, for their expert histopathological workup of the study specimens.

# References

1. Schneider M, Strobel O, Hackert T, Büchler MW. Pancreatic resection for cancer – the Heidelberg technique. Langenbecks Arch Surg. 2019;404(8):1017–1022. https://doi.org/10.1007/s00423-019-01839-1

2. Klotz R, Hackert T, Heger P, Probst P, Hinz U, Loos M, et al. The TRIANGLE operation for pancreatic head and body cancers: early postoperative outcomes. HPB (Oxford). 2022;24(3):332–341. https://doi.org/10.1016/j.hpb.2021.06.432

3. Hang HX, Cai ZH, Yang YF, Fu X, Qiu YD, Cheng H. Comparison of prognosis and postoperative morbidities between standard pancreaticoduodenectomy and the TRIANGLE technique for resectable pancreatic ductal adenocarcinoma. World J Gastrointest Surg. 2024;16(3):689–699. <https://doi.org/10.4240/wjgs.v16.i3.689>

4. Lei K, Wang J, Wang X, Wang H, Hu R, Zhang H, et al. Extended lymphadenectomy based on TRIANGLE for pancreatic head cancer: a single-center experience. Langenbecks Arch Surg. 2024;409(1):54. https://doi.org/10.1007/s00423-024-03245-8

5. Luzon JA, Thorsen Y, Nogueira LP, Andersen SN, Edwin B, Haugen HJ, et al. Reconstructing topography and extent of injury to the superior mesenteric artery plexus in right colectomy with extended D3 mesenterectomy: a composite multimodal 3-dimensional analysis. Surg Endosc. 2022;36(10):7607-7618. doi: 10.1007/s00464-022-09200-2.

6. Thorsen Y, Stimec BV, Lindstrom JC, Oresland T, Ignjatovic D. Stool dynamics after extrinsic nerve injury during right colectomy with extended D3-mesenterectomy. Scand J Gastroenterol. 2021;56(7):770–776. <https://doi.org/10.1080/00365521.2021.1918757>

7. Chen JH, Zhu LY, Cai ZW, Hu X, Ahmed AA, Ge JQ, et al. TRIANGLE operation, combined with adequate adjuvant chemotherapy, can improve the prognosis of pancreatic head cancer: a retrospective study. World J Gastrointest Oncol. 2024;16(5):1773–1786. https://doi.org/10.4251/wjgo.v16.i5.1773
